# Supplementary figures and images for: Regulation of DNA methylation on key parasitism genes of Cysticercus cellulosae revealed by integrative epigenomic-transcriptomic analyses
Source: Hereditas. 2021 Aug 12;158:28. doi: 10.1186/s41065-021-00195-9 (PMC8361615; doi:10.1186/s41065-021-00195-9)

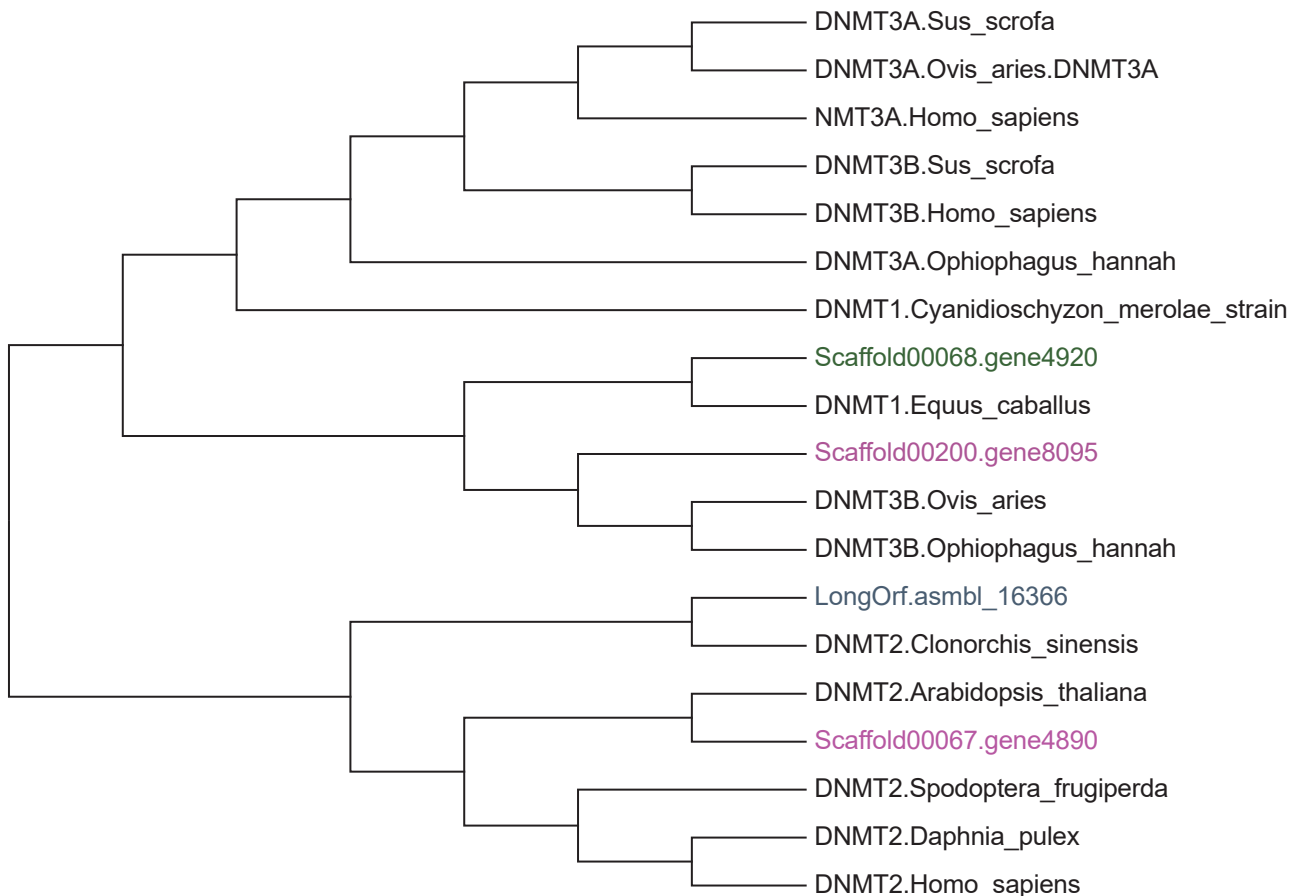

Supplement: Supplementary file 1 — Additional file 1: Figure S1. Phylogenetic tree of DNMT proteins. [file 41065_2021_195_MOESM1_ESM.pdf]

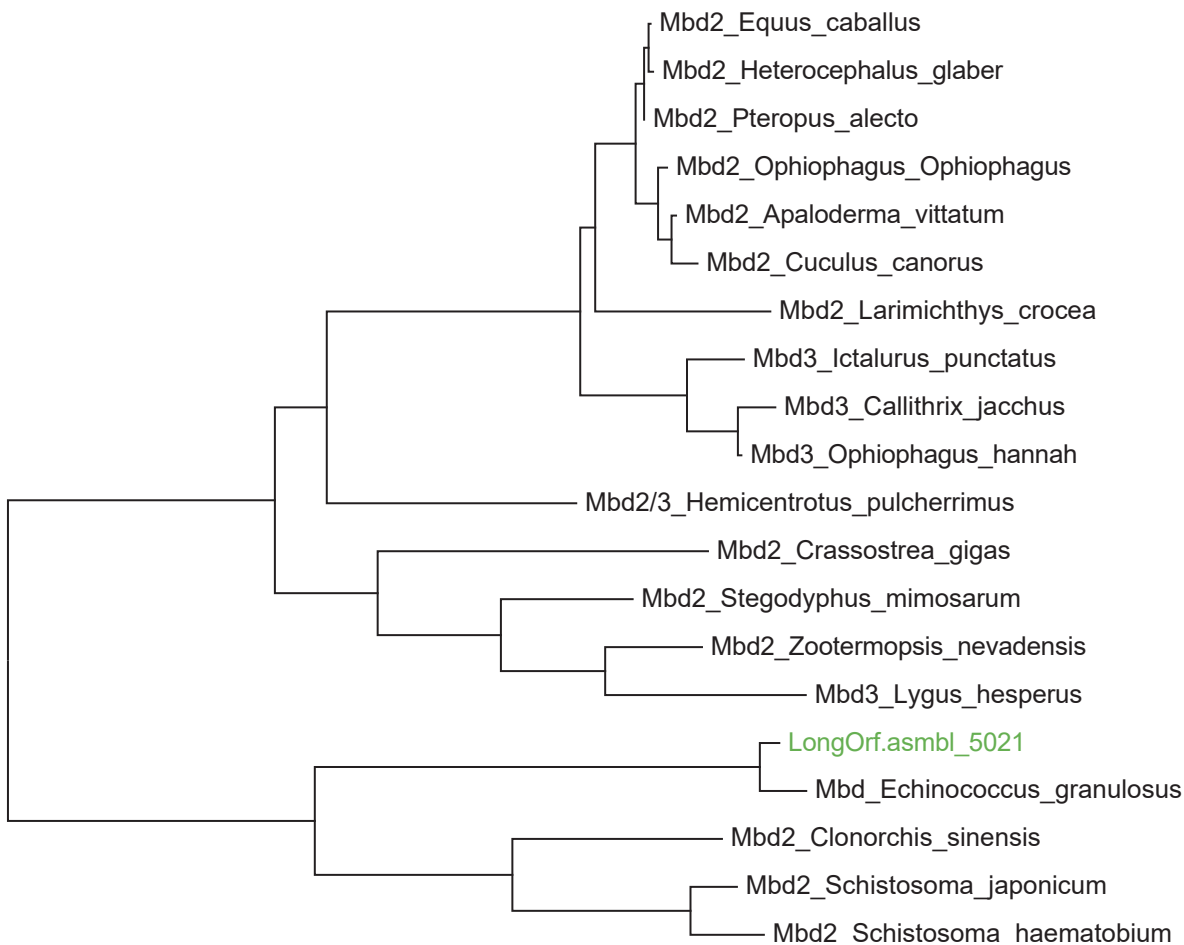

0.20

Supplement: Supplementary file 2 — Additional file 2: Figure S2. Phylogenetic tree of mbd proteins. [file 41065_2021_195_MOESM2_ESM.pdf]

(A)

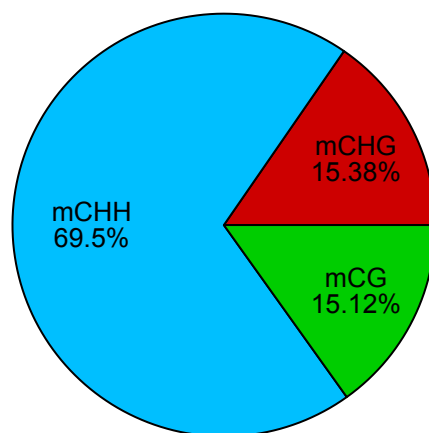

(B)

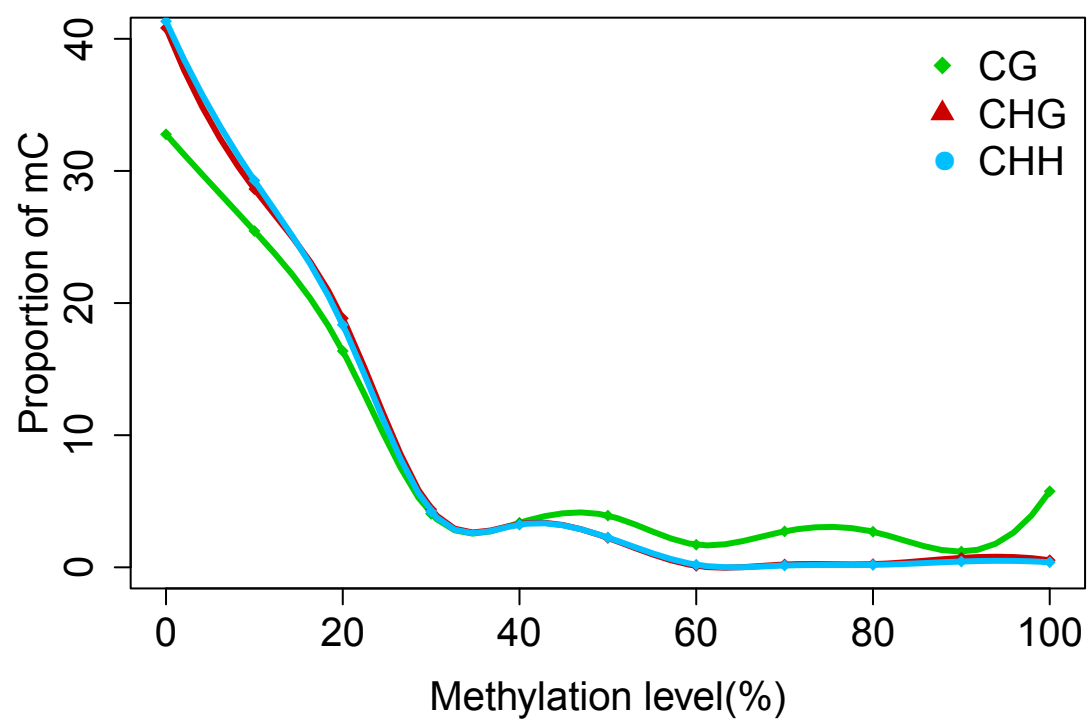

(C)

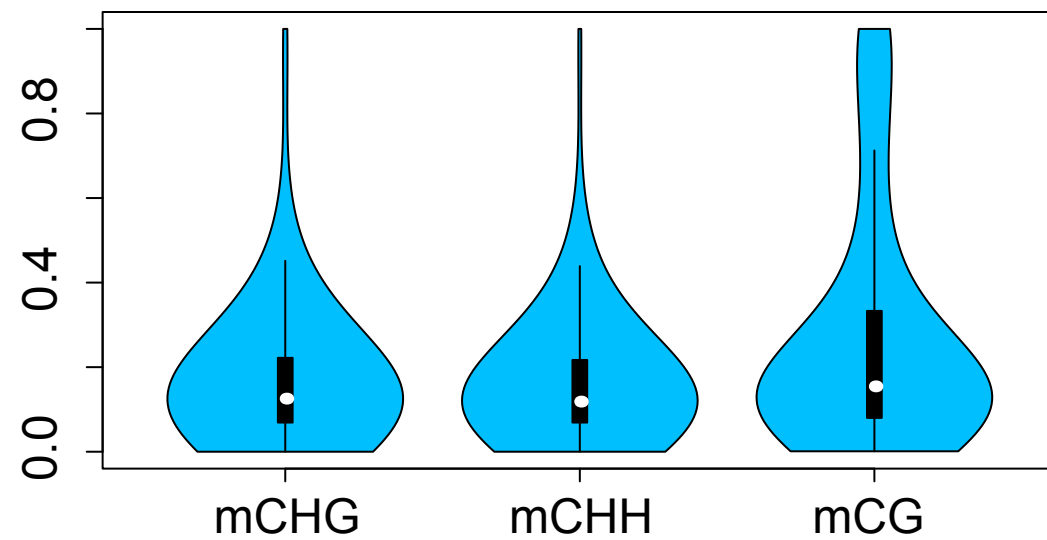

(D)

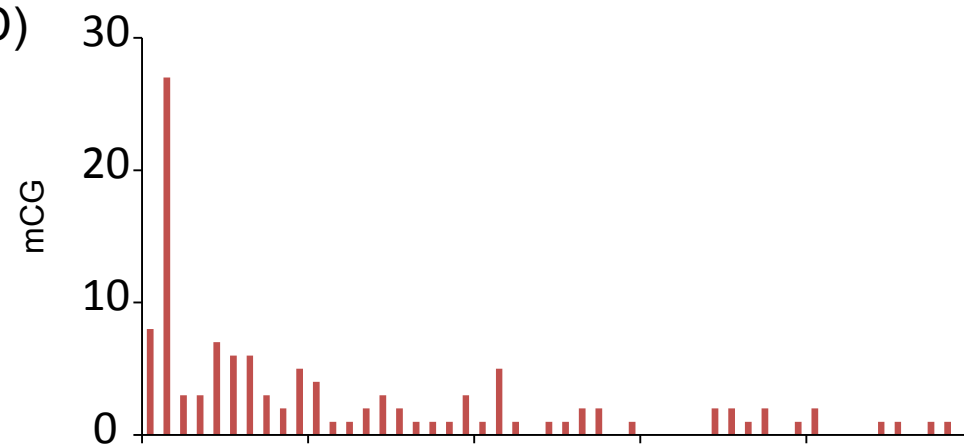

(E)

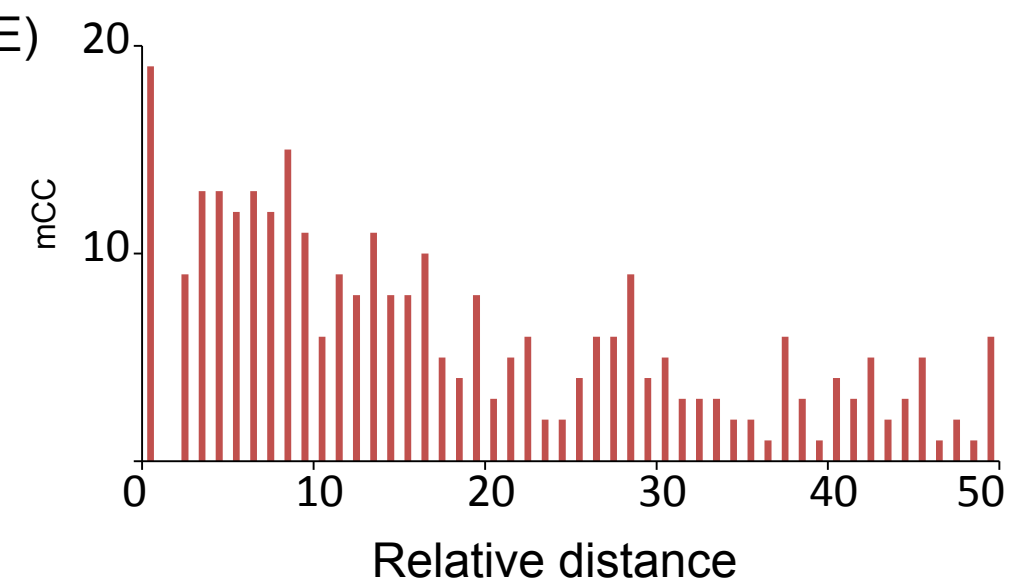

Supplement: Supplementary file 3 — Additional file 3: Figure S3. Patterns and chromosomal distribution of DNA methylation in T. solium. [file 41065_2021_195_MOESM3_ESM.pdf]

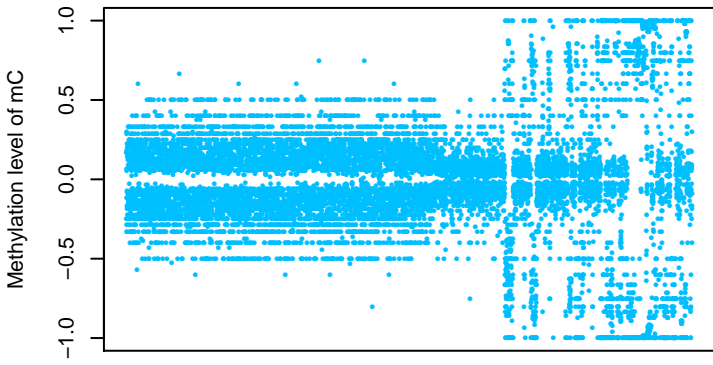

mCG on genome

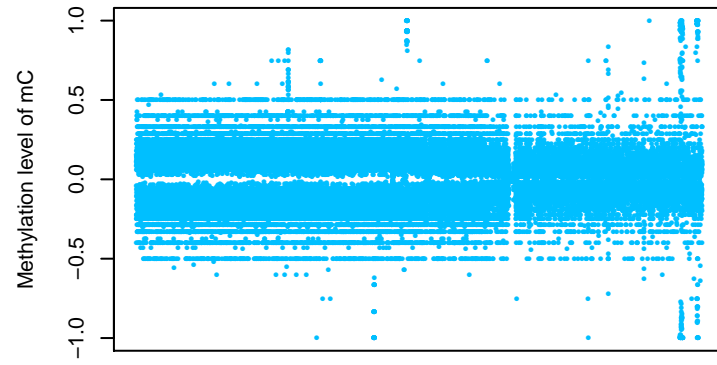

mCA on genome

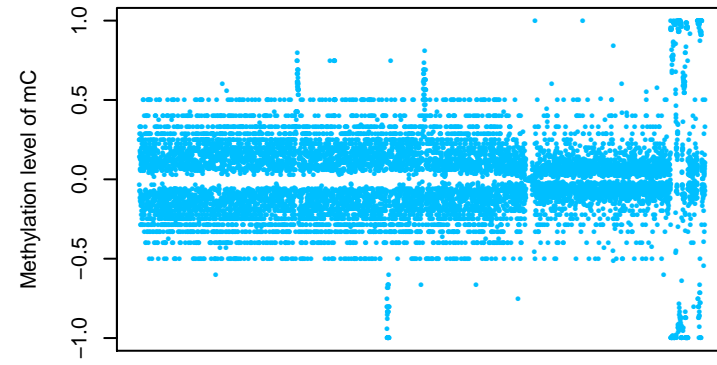

mCC on genome

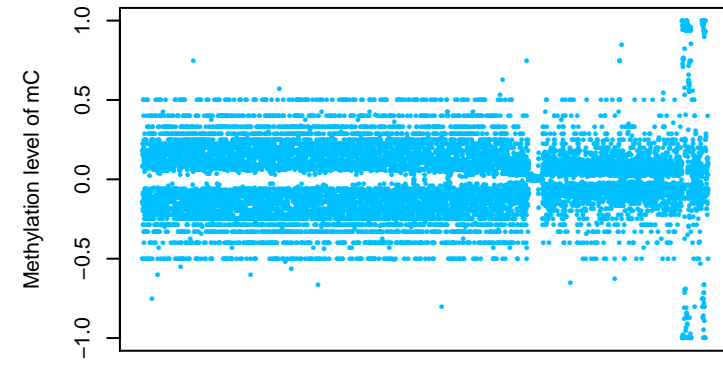

mCT on genome

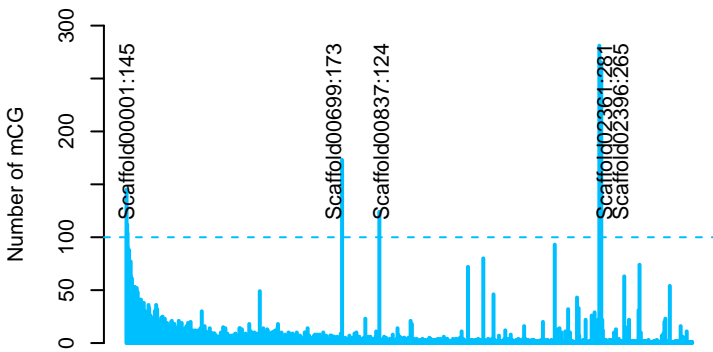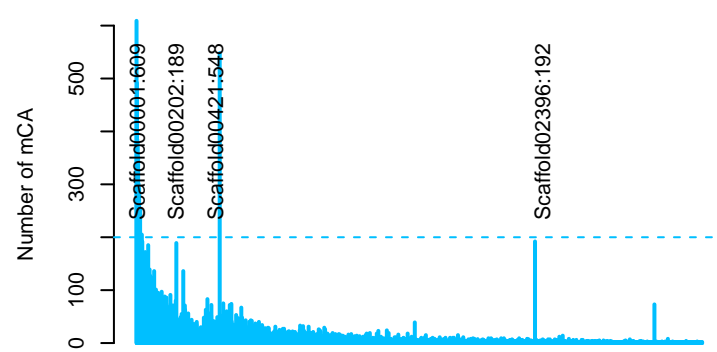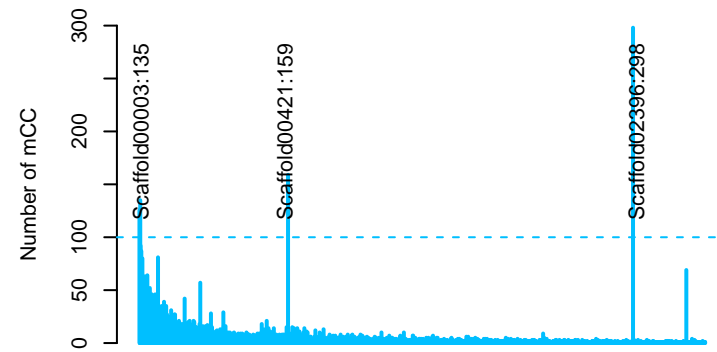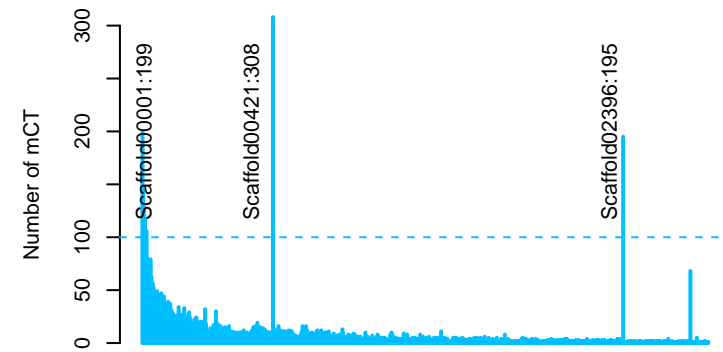

Supplement: Supplementary file 4 — Additional file 4: Figure S4. DNA methylation patterns and chromosomal distribution. [file 41065_2021_195_MOESM4_ESM.pdf]

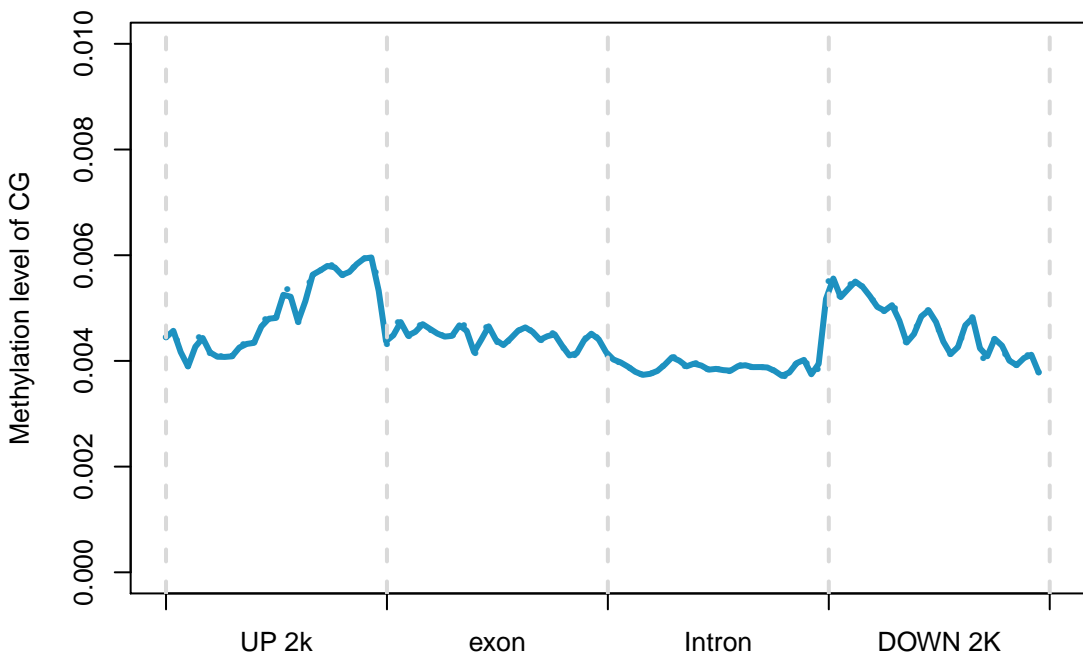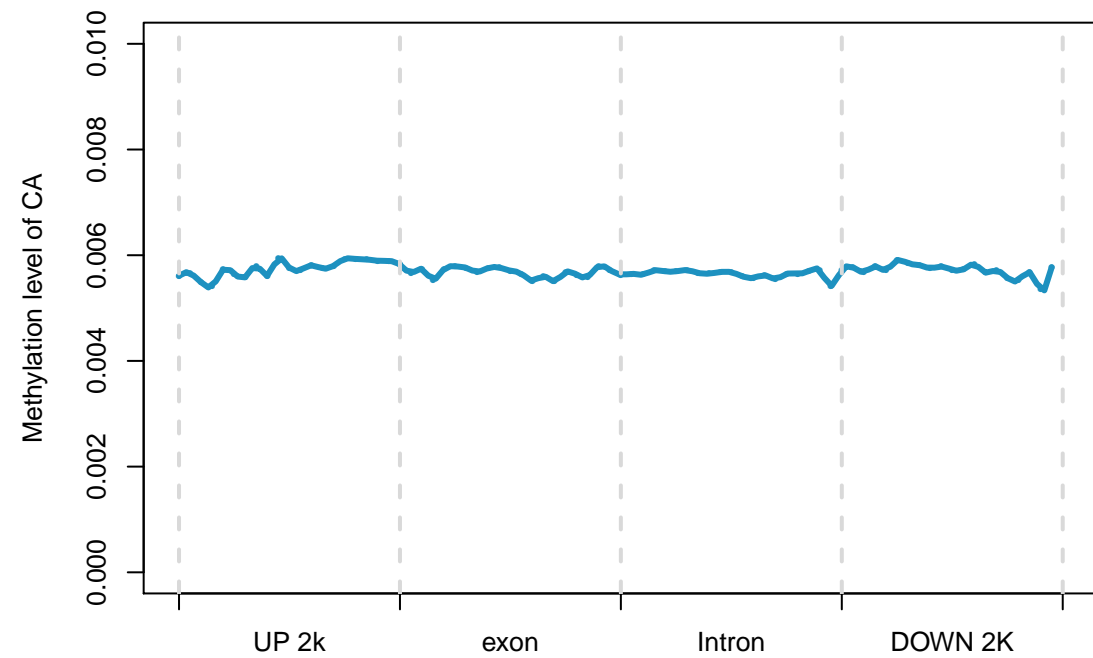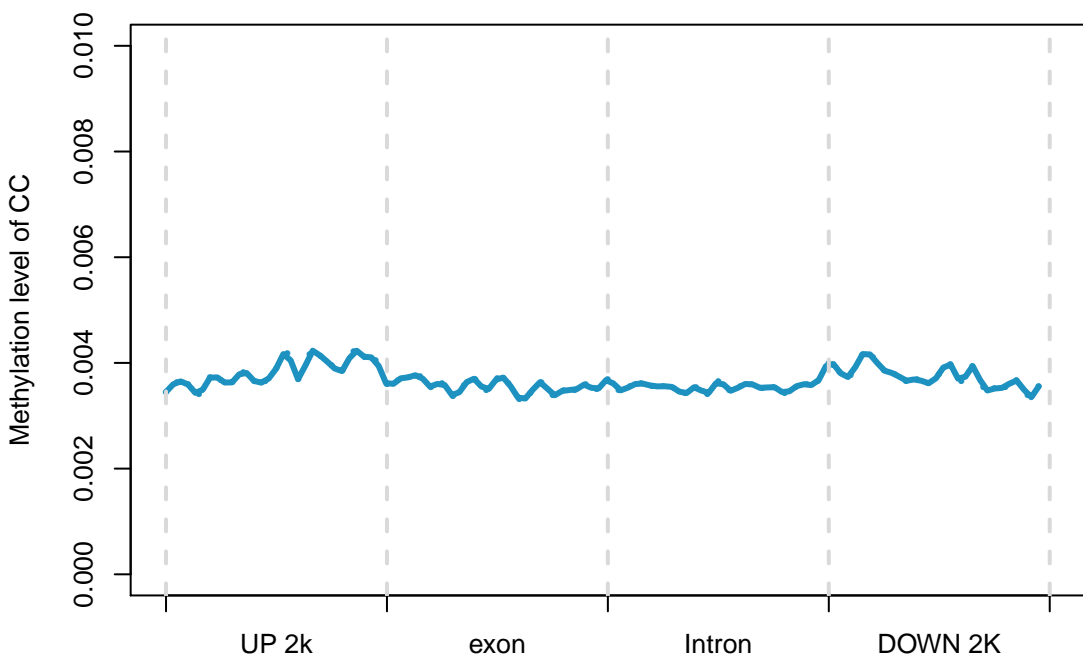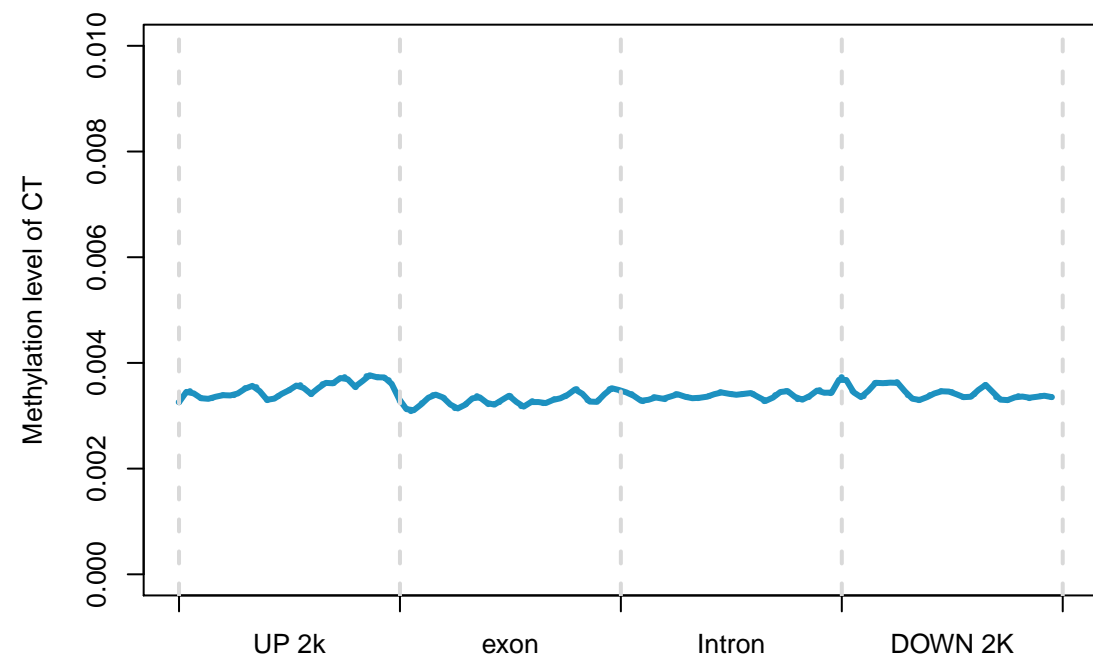

Supplement: Supplementary file 5 — Additional file 5: Figure S5. Average density of methylation levels of cytosine distributed on genome. [file 41065_2021_195_MOESM5_ESM.pdf]

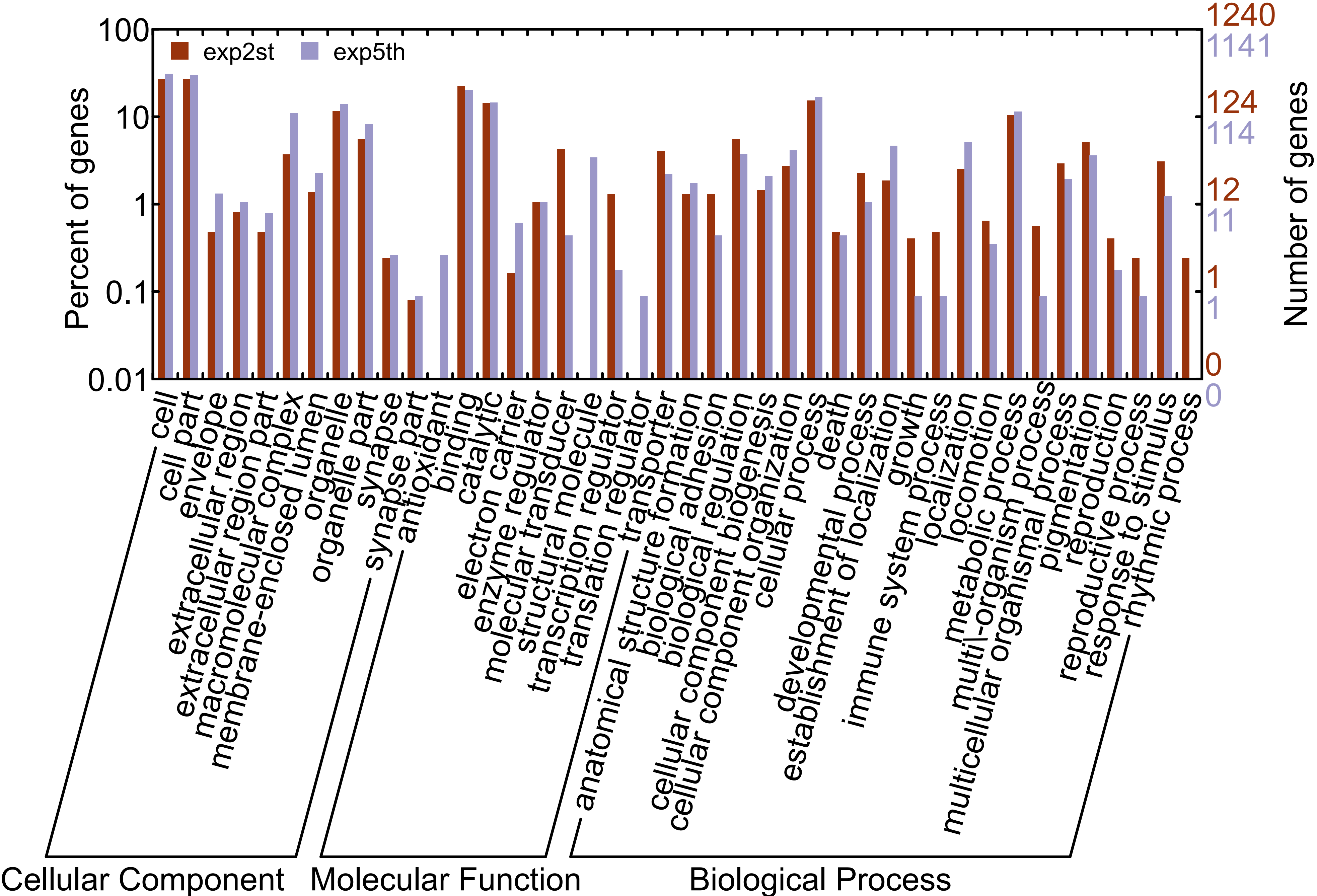

Supplement: Supplementary file 6 — Additional file 6: Figure S6. Gene Ontology (GO) analysis for the genes with the lowest expression (2nd) and the most highly expressed (5th) genes. [file 41065_2021_195_MOESM6_ESM.pdf]
